# Elevated corticosterone levels decrease reproductive output of chick-rearing Adélie penguins but do not affect chick mass at fledging

**DOI:** 10.1093/conphys/cot007

**Published:** 2013-05-14

**Authors:** Anne-Mathilde Thierry, Yan Ropert-Coudert, Thierry Raclot

**Affiliations:** 1 Université de Strasbourg, IPHC, 23 rue Becquerel, 67087 Strasbourg, France; 2 CNRS, UMR7178, 67037 Strasbourg, France

**Keywords:** Breeding effort, glucocorticoids, *Pygoscelis adeliae*, reproductive output, seabird

## Abstract

Study of physiological mechanisms can help us to understand how animals respond to changing environmental conditions. In particular, stress hormones (i.e. glucocorticoids, such as corticosterone) are described as mediating resource allocation, allowing animals to adjust their physiology and behaviour to predictable and unpredictable changes in the environment. In this study, we investigated the effects of an experimental increase in baseline corticosterone levels on the breeding effort and the reproductive output of chick-rearing male Adélie penguins (*Pygoscelis adeliae*). The number of chicks per nest, their body mass, and their size were monitored throughout the study. Direct observations allowed measurement of the time spent foraging at sea and caring for the young on the nest. At the end of the treatment, blood samples were collected for isotope analysis. Although all birds raised at least one chick, reproductive output was decreased by 42% in corticosterone-treated birds compared with control birds. The increase in corticosterone levels during the guard stage did not affect the mass of surviving chicks or the brood mass at fledging. Corticosterone-treated males spent on average 21% more time at the nest than control birds. However, the duration of foraging trips was similar between both groups. In addition, the similarity of isotopic signatures suggests that both groups foraged at similar locations and ingested the same prey species. The detailed on-land behaviour of birds should be examined in further studies to clarify the possible links between corticosterone levels, brooding time, and reproductive output. Understanding the relationships between glucocorticoids, fitness, and ultimately population dynamics is fundamental to enabling conservation physiology as a discipline to be successful in helping to manage species of conservation concern.

## Abstract (precis)

Stress hormones allow animals to adjust their physiology and behaviour to predictable and unpredictable changes in the environment. We investigated the effects of an increase in stress hormone levels on the breeding effort and the reproductive ouput of chick-rearing male Adélie penguins.

## Introduction

Organisms live in a changing environment, which they deal with by adjusting their morphology, physiology, and behaviour to face current conditions. Many studies report that increases in environmental variability associated with climate change affect wildlife drastically (Walther *et al.*, 2002; Stenseth *et al.*, 2003). For instance, cases of animal population decline in response to such changes are increasingly reported (McCarthy *et al.*, 2001; Croxall *et al.*, 2002; Both *et al.*, 2006). Animals face trade-offs in terms of how they allocate energy to different biological functions, such as reproduction and survival (Stearns, 1992). Although changes in key life-history trade-offs are thought to be at the heart of these population declines (e.g. Woodhams *et al.*, 2008; Acevedo-Whitehouse and Duffus, 2009), we still know little about the mechanisms underlying these declines. In order to achieve a better understanding of how organisms respond to changing environmental conditions, physiological mechanisms must be considered (Pörtner, 2002; Chown and Gaston, 2008; Pörtner and Farrell, 2008; Chown *et al.*, 2010; Fuller *et al.*, 2010). In particular, stress hormones (i.e. glucocorticoids) are described as mediating resource allocation, allowing animals to adjust their physiology and behaviour to both predictable and unpredictable regimens of environmental variations (Jacobs and Wingfield, 2000; Wingfield and Silverin, 2009).

Glucocorticoids are acutely released during life-threatening situations, such as food shortage (Kitaysky *et al.*, 1999), severe weather conditions (Romero *et al.*, 2000), and acute predation risk (Cockrem and Silverin, 2002). The activation of the hypothalamic–pituitary–adrenal axis and the subsequent release of glucocorticoids trigger the emergency life-history stage, i.e. when an individual aborts the current breeding attempt in order to survive the perturbation (Wingfield *et al.*, 1998; Wingfield and Kitaysky, 2002; Wingfield, 2003; Landys *et al.*, 2006). Elevated glucocorticoid levels can affect the physiology and behaviour of animals in a variety of ways. In particular, stress hormones control energy metabolism and fuel utilization and may promote escape behaviour through glucose mobilization and increased locomotor activity, finally leading to a reduction in or abandonment of the reproductive effort (see Landys *et al.*, 2006; Breuner, 2011 for review). Elevated glucocorticoid levels also enhance activities related to foraging behaviour and food intake.

In birds, elevated baseline levels of corticosterone (CORT; the main avian glucocorticoid) generally observed during reproduction might facilitate reproductive effort (Romero, 2002; Love *et al.*, 2004), especially allowing individuals to supply their offspring through its positive effect on foraging activity (Koch *et al.*, 2002, 2004; Angelier *et al.*, 2007, 2008; Miller *et al.*, 2009; Crossin *et al.*, 2012).

On the contrary, high CORT levels are suspected to disrupt parental behaviour, because they are often associated with abandonment of reproduction in birds (Silverin, 1986; Wingfield and Sapolsky, 2003; Groscolas *et al.*, 2008; Spée *et al.*, 2010). These contrasting effects seem to be driven by extrinsic factors; during unfavourable environmental conditions, when organisms cope with high energetic constraints, CORT could redirect energy allocation from the provisioning of chicks to the benefit of self-maintenance (reviewed by Wingfield *et al.*, 1998). Furthermore, it is often assumed, despite little direct evidence, that the acute adrenocortical response to stress favours self-maintenance behaviour at the expense of current reproduction (see Breuner *et al.*, 2008 for a detailed review of the relationships between the acute adrenocortical response and fitness). Growing evidence suggests that the modulation of baseline CORT levels participates in the mediation of trade-offs between current reproductive output (parental investment) and self-maintenance through foraging activities (Kitaysky *et al.*, 2001; Landys *et al.*, 2006; Angelier *et al.*, 2007, 2008; Horton and Holberton, 2009).Subcutaneous CORT implants that modulate baseline CORT levels may lead to better understanding of the mechanisms that link glucocorticoids and reproductive effort, and hence allow the establishment of conservation measures in species facing changes in their environment.

Seabirds respond to food shortages by an increase in the circulating baseline corticosterone levels. For example, a 3-fold increase in stress hormone levels was measured in food-deprived black-legged kittiwake (*Rissa tridactyla*) adults and chicks (Kitaysky *et al.*, 2001). An artificial increase in the CORT level could, to some extent, mimic the effect of an environmental stressor. The objectives of this study were, therefore, to examine the consequences of an experimental increase in baseline CORT levels on the parental effort and reproductive output of control and CORT-treated Adélie penguins (*Pygoscelis adeliae*).

Polar ecosystems are relatively pristine environments (Bargagli, 2004). Yet, recent climate change poses a new challenge to the survival of Arctic and Antarctic wildlife. For example, Adélie penguin populations are increasing in the Ross Sea region and decreasing in the Antarctic Peninsula, with an overall increase of the net global population (Ainley *et al.*, 2010). However, the species is expected to undergo a 30% population decline over the next three generations due to the effects of projected climate change, in particular in association with a decrease in the concentration of sea ice (Ainley *et al.*, 2010). Loss of sea ice can be seen as a major stressor for Adélie penguins and other top predators. Indeed, sea ice is the preferred habitat of Antarctic krill (*Euphausia superba*), the main food source of penguins, leading to a strong dependence of Adélie penguins on sea ice (for discussion of Adélie penguins as a ‘creature of the pack ice’, see Ainley, 2002). There can be important interannual variations in environmental conditions in Antarctica, in particular regarding the extent of sea-ice and the timing of its retreat. These changes can have major consequences for the breeding success of Adélie penguins (Emmerson and Southwell, 2008), their corticosterone levels (Cockrem *et al.*, 2006), and the durations of their foraging trips (Beaulieu *et al.*, 2010). The species has recently been uplisted from Least Concern to Near Threatened on the IUCN Red List for these reasons (BirdLife International, 2012). Future climatic changes remain largely uncertain, and further work is required to determine how they will impact penguins. As such, we rapidly need to establish a benchmark for future investigations and to understand the factors that affect the breeding success of Adélie penguins.

Exogenous CORT induced nest abandonment of fasting, incubating male Adélie penguins (Spée *et al.*, 2011a), together with decreased incubation temperatures and a lengthened incubation period (Thierry *et al.*, 2013). We would therefore expect CORT treatment to increase the rate of nest desertion of chick-rearing male Adélie penguins, resulting in decreased reproductive output. In this case, individuals would tend to allocate their energy to self-maintenance at the expense of current reproduction. In contrast, CORT implants in female macaroni penguins (*Eudyptes chrysolophus*) were found to affect foraging behaviour, parental care, and chick growth in a positive manner (Crossin *et al.*, 2012). Consequently, positive effects of CORT treatment on reproductive effort could also be expected in our study. In order to distinguish between these contrasting predictions, we manipulated the CORT levels and examined the effects of the treatment on the parental effort of chick-rearing Adélie penguins.

## Materials and methods

### Study site and species

The study was carried out at the French research station Dumont d'Urville (66°40′S, 140°01′E), East Antarctica, during the 2008–2009 austral summer. Adélie penguins reproduce once a year. Their breeding cycle comprises four distinct stages from mid-October to mid-February: courtship, incubation, guard stage, and crèche stage. This study focuses on the guard stage, when both parents alternate between foraging at sea and chick attendance at the nest.

Adélie penguins weigh 3.2–8 kg, depending on the life-history stage. Females usually lay two eggs (mean clutch size, 1.8), and 1.6 chicks per nest hatch. About one chick is fledged per breeding pair, with a mean weight at fledging of ∼3 kg (Ainley, 2002). Although in Adélie penguins, as in most seabird species, both parents care for their young, previous studies on stress hormones in Adélie penguins have mostly considered male birds (Spée *et al.*, 2011a; Thierry *et al.*, 2013) because males can fast for up to 40–50 days at the beginning of the breeding season (Vleck and Vleck, 2002). In order to obtain data comparable with these previous studies and because treating both partners could induce confounding effects or be deleterious for the current reproduction, only male Adélie penguins were studied here.

### Study protocol

The protocol was approved by the ethics committee of the French Polar Institute (Institut Paul-Emile Victor; IPEV) and authorized by the French Southern and Antarctic Territories (Terres Australes et Antarctiques Françaises; TAAF).

Thirty randomly selected pairs were captured on their nest at the end of the courtship (mid-November). Each member of the pair was identified with a Nyanzol-D number painted on the chest feathers and stickers inserted between the back feathers (Beaulieu *et al.*, 2010), allowing easy identification in the colony. Penguins were sexed by a combination of parameters, including cloacal inspection before egg laying and observations of incubation routine (Kerry *et al.*, 1993; Beaulieu *et al.*, 2010). Visual observations of the 30 nests were made from a distance every 2–3 h each day during the entire study period, in order to observe laying, hatching, and presence of each partner on the nest, and to measure foraging trip duration.

**Figure 1:**
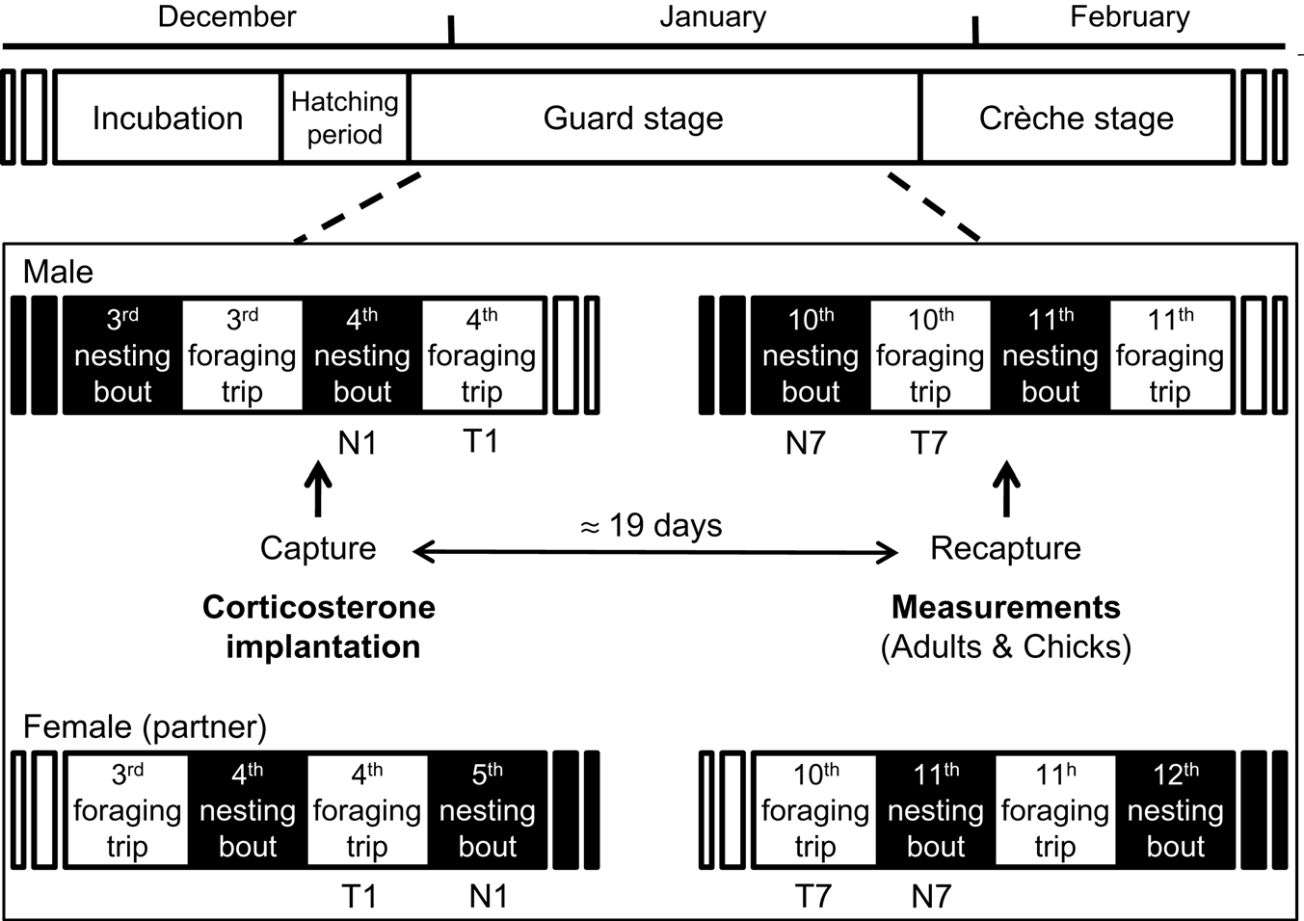
breeding phenology of Ade^'^lie penguins and study protocol during the chick-rearing period (guard stage). The studied male penguins were captured twice and monitored throughout this period.

At the beginning of the guard stage, pairs were randomly assigned to control (*n* = 7) and experimental groups (*n* = 7) among the 30 pairs marked during the courtship, which were synchronized in their breeding cycle (similar hatching dates and number of foraging trips made before implantation). All males were captured on two occasions (see Fig. 1). To minimize stress, a bird's head was covered with a hood (Cockrem *et al.*, 2008) and chicks were kept safe.

All 14 males were captured at the beginning of the guard stage (28 December–2 January). Half of them (experimental group) were implanted with a self-degradable CORT-releasing pellet, while the others (control group) underwent the same procedure without implantation of a pellet. For both groups, a small area of skin around the nape of the neck was disinfected with 70% alcohol, an incision ∼1 cm long was made, and this incision was finally (after implantation for the experimental group) closed with a sterile stitch and sprayed with Alumisol^®^ (healing external suspension). Corticosterone pellets (100 mg, 21 days release; Innovative Research of America, Sarasota, FL, USA) were implanted subcutaneously in the nape of the neck. Previous studies had shown that these pellets led to a 2- to 4-fold increase of CORT levels in free-living male Adélie penguins (Spée *et al.*, 2011a; Thierry *et al.*, 2013).

At the end of the guard stage/early crèche stage, 19 ± 2 days after the first capture (16–19 January), males returning from a foraging trip were recaptured together with their chicks. A small blood sample (∼0.3 mL) was collected from the alar or tarsal vein and subsequently kept at −20°C until stable isotope analysis. For 4 (3 control group chicks and 1 CORT group chick) of the 18 chicks (12 control group chicks and 6 CORT group chicks), blood sampling was not performed due to severe weather conditions during the sampling period. A control pair placed next to a crèche had no attributable chick, so that only 13 chicks were included for blood analyses. The mass and flipper length of adult males and chicks were measured using a Pesola spring balance (5 kg ± 0.3% for chicks and 10 kg ± 0.3% for adults) and a ruler (± 1 mm), respectively. In adult penguins, the flipper length has been considered to provide a good indicator of body size, because flippers do not grow after fledging (Mínguez *et al.*, 1998). A scaled mass index was calculated as previously reported (Peig and Green, 2009). Chicks were also weighed and measured 39–43 days after hatching, when their weight is at a maximum (Ainley, 2002).

### Reproductive output

During the study period, the number of chicks was checked thoroughly (by gently pushing the adult present on the nest when needed) on several occasions: before treatment (27 December), at capture devoted to CORT implantation or sham manipulation, during treatment (9 January), at the end of the study during recapture, and 39–43 days after hatching. This was done to assess the reproductive output, defined as the number of chicks per nest, the body weight of the chicks, and the brood mass.

### Stable isotope analyses

In Adélie Land, penguins are known to feed principally on a mix of krill and fish (Wienecke *et al.*, 2000). The stable isotope signatures have been evaluated by Cherel (2008) for Antarctic krill (*E. superba*; δ^13^C = −25.4 ± 0.6, δ^15^N = 5.3 ± 0.5; sampled in summer 2002), ice krill (*Euphausia crystallorophias*; δ^13^C = −25.4 ± 0.4, δ^15^N = 6.8 ± 0.7; sampled in summer 2002), and Antarctic silverfish (*Pleuragramma antarcticum*; δ^13^C = −24.7 ± 0.4, δ^15^N = 10.6 ± 0.3, sampled in winter/spring 2002). The stable isotope analysis of the diet of Adélie penguins is known to be relatively consistent with analyses of prey found in their stomach content (Tierney *et al.*, 2008).

The tissue isotopic signature mirrors the diet throughout the period of tissue synthesis (Bearhop *et al.*, 2002), and according to Cherel & Hobson (2007), whole blood has a 1 month turnover in large birds. Thus, we assume that our isotopic measure integrates the diet of adult males over the whole treatment period. Given that chicks are unable to feed by themselves, their isotopic signature depends largely on the food brought by their parents. Stable carbon and nitrogen assays were carried out at the Centre de Recherche sur les Ecosystèmes Littoraux Anthropisés, L'Houmeau, France. Replicate measurements showed coefficients of variation for δ^13^C and δ^15^N values of standard acetanilide of 0.34 and 9.61%, respectively. Values are expressed in the usual δ notation (‰) relative to Pee Dee Belemnite (PDB) for δ^13^C and atmospheric nitrogen (N_2_) for δ^15^N.

### Data analysis

All statistical analyses were performed with R 2.13.2 (R Development Core Team, 2011). Results are expressed as means ± SEM. Differences were considered statistically significant when *P* < 0.05.

Owing to the small sample sizes, Wilcoxon tests were used to compare adult male body mass, scaled mass index, hatching date between groups, initial and final capture dates, and the number of chicks per pair before and at the end of the treatment (control *vs.* CORT).

Generalized estimating equations (GEE) were used to compare time budget (time spent at sea and time spent at the nest), reproductive output (number of chicks per nest), and chick mass between control birds and CORT-treated birds. Generalized estimating equations were computed using the *geeglm* function of the *geepack* package in R (Højsgaard *et al.*, 2006). Model selection was performed by excluding non-significant interactions first, and then all non-significant factors. Statistics and *P*-values of non-significant factors and/or interactions are reported before removal from the model.

Regarding time budget analysis, the first nesting bout (N1) of males and the first foraging trip of females (T1) of the experiment were removed from analyses because first capture and treatment were carried out during this period. In addition, to homogenize the number of individuals per group, only the first seven foraging trips after treatment were considered for the analyses (see Fig. 1), i.e. during the time that the treatment was efficient. Treatment, foraging trip (T1, T2, etc.) or nesting bout (N1, N2, etc.) ranks, and the interaction between treatment and foraging trip rank or between treatment and nesting bout rank were taken into account as fixed factors. Data obtained from males and females (partners) were analysed separately because only males were CORT treated. Wilcoxon tests were used to compare the number of foraging trips made between 1 and 15 days after implantation and between 1 and 21 days after implantation, and the total time spent at the nest during these periods between groups.

Relationships between adult mass (or scaled mass index) and brood mass at the end of the treatment were estimated with Spearman correlation tests.

Multivariate analysis of variance (MANOVA) with Wilk's lambda statistics was used to compare overall isotopic signatures between groups, according to the condition of residuals normality (assessed by Shapiro–Wilk test). Differences in δ^13^C and δ^15^N between control males and their chicks and between CORT-treated males and their chicks were assessed with Wilcoxon tests.

## Results

There was no difference in the date when birds were captured and treated (control *vs.* CORT) at the beginning of the chick-rearing period (Wilcoxon, *W* = 29, *P* = 0.627). Control and CORT-treated birds were recaptured on similar dates at the end of the guard stage/early crèche stage (*W* = 17, *P* = 0.333).

### Time budget

**Figure 2:**
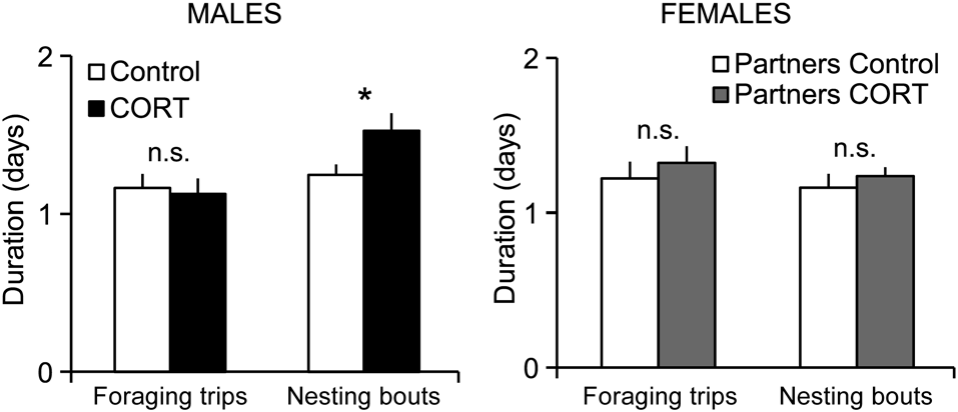
mean duration (±SEM) over the study period of foraging trips and nesting bouts for corticosterone (CORT)-treated and control male Adélie penguins and their partners (n = 7 per group). *Significant difference (P < 0.05) between the two treatments; n.s., not significant.

**Figure 3:**
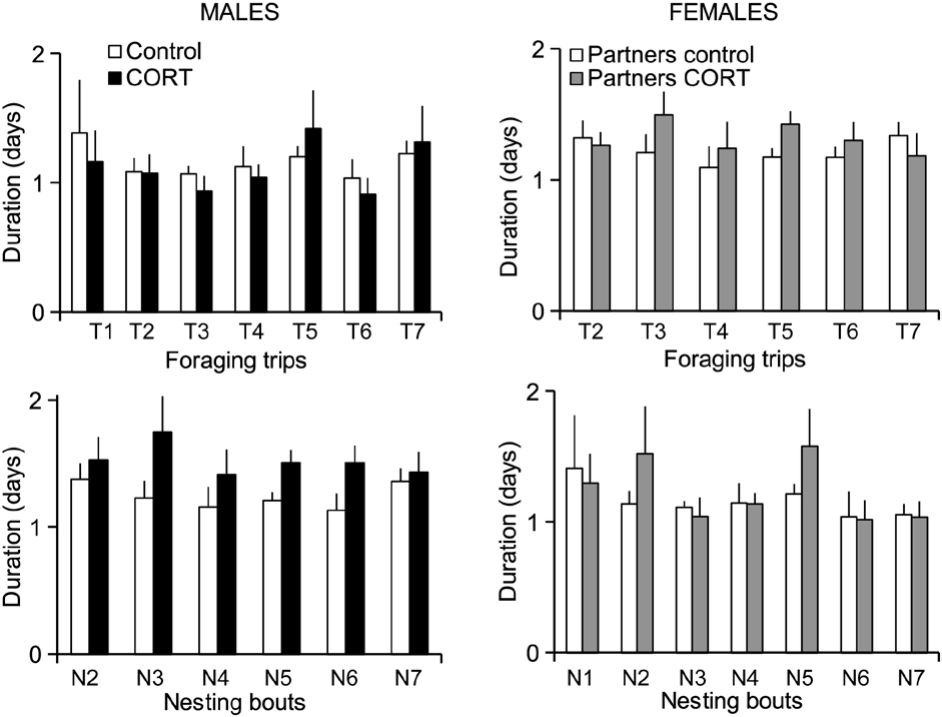
duration (means ± SEM) of successive foraging trips and nesting bouts (following the experimental treatment) for treated and control male Adélie penguins and their partners (n = 7 per group).

The time budget of control birds was similar between sexes, with foraging trips (Generalised Estimating Equations, Wald χ^2^ = 0.496, *P* = 0.481) and nesting bouts (Wald χ^2^ = 1.47, *P* = 0.225) lasting on average 1.2 days for both males and females (Figs 2 and 3).

For males, time spent at sea was not affected by the treatment (Fig. 2; Wald χ^2^ = 0.008, *P* = 0.930), the foraging trip rank (Wald χ^2^ = 0.707, *P* = 0.400), or the interaction treatment × foraging trip rank (Wald χ^2^ = 0.733, *P* = 0.733). However, nesting bouts were significantly longer in CORT-treated males compared with control birds (Wald χ^2^ = 4.747, *P* = 0.029; Fig. 2). Neither the nesting bout rank (Wald χ^2^ = 0.113, *P* = 0.737) nor the interaction treatment × nesting bout rank (Wald χ^2^ = 0.849, *P* = 0.357) had a significant effect on the time spent on the nest. Consequently, the number of foraging trips tended to be lower, although not significantly so, in CORT-treated birds compared with control birds (5.9 ± 0.3 *vs.* 6.6 ± 0.3 foraging trips, respectively, after 15 days, *W* = 11.5, *P* = 0.087; and 8.1 ± 0.4 *vs.* 9.1 ± 0.5, respectively, after 21 days, *W* = 13.5, *P* = 0.169). The CORT-treated males spent on average 21% more time at the nest and 17% less time at sea than control males between 1 and 15 days after pellet implantation (8.6 ± 0.5 days at the nest *vs.* 7.1 ± 0.4 days for CORT-treated birds and controls, respectively, *W* = 40, *P* = 0.055; and 7.6 ± 0.3 *vs*. 6.3 ± 0.4 days days at sea, respectively, *W* = 9, *P* = 0.055).

For females, the time spent on the nest was not affected by the treatment of their mates (Wald χ^2^ = 1.637, *P* = 0.201), the nesting bout rank (Wald χ^2^ = 0.944, *P* = 0.331), or the interaction of these two factors (Wald χ^2^ = 0.357, *P* = 0.550). In addition, female foraging trip duration was not affected by the partner treatment (Wald χ^2^ = 0.902, *P* = 0.342), the foraging trip rank (Wald χ^2^ = 0.105, *P* = 0.746), or the interaction of partner treatment × foraging trip rank (Wald χ^2^ = 1.779, *P* = 0.182).

Given that CORT-treated males spent more time at the nest while their partners did not perform longer trips, treated pairs spent more time together at the nest (Wald χ^2^ = 8.901, *P* = 0.003). The nesting bout rank (Wald χ^2^ = 0.000, *P* = 0.988) and the interaction treatment × nesting bout rank (Wald χ^2^ = 0.002, *P* = 0.966) had no significant effect on the time spent together at the nest.

### Reproductive output and body condition

**Figure 4:**
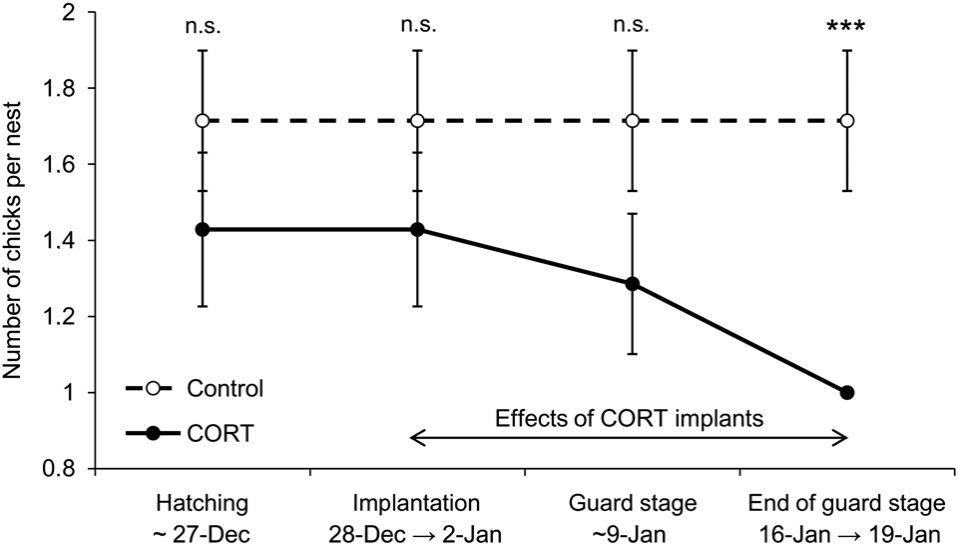
reproductive output of Adélie penguin pairs according to experimental treatment throughout the study period. Values are means ± SEM. ***Significant difference (*P* < 0.001) between the two treatments; n.s., not significant.

There was no difference in the number of chicks per pair between control and CORT-treated birds at the beginning of the experiment (*W* = 31.5, *P* = 0.334). The CORT treatment resulted in a significant 42% decrease in the number of chicks per nest (Wald χ^2^ = 9.122, *P* = 0.003). Neither the period during which the number of chicks was checked (Wald χ^2^ = 2.047, *P* = 0.152) nor the interaction period × treatment (Wald χ^2^ = 2.637, *P* = 0.104) affected this parameter (Fig. 4). At the end of the experiment, all birds were successful breeders, but CORT-treated males raised only one chick, while control birds raised the same number of chicks as at the beginning of the experiment (*W* = 42, *P* = 0.009).

**Figure 5:**
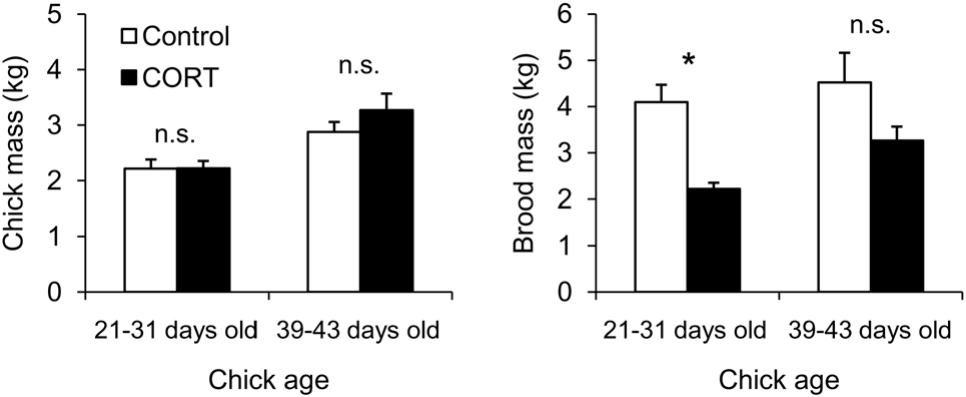
chick body mass (left panel) and brood mass (right panel) of control and CORT-treated Adélie penguin pairs according to chick age throughout the study period. Values are means ± SEM. *Significant difference (*P* < 0.05) between the two groups; n.s., not significant.

No differences in the body mass and scaled mass index of the adult males were observed between the two groups (Table 1), and the CORT treatment did not induce nest abandonment. As a result of the greater chick mortality, CORT-treated birds had to feed a 42% lower brood mass compared with control birds at the end of the experiment (brood mass in CORT group = 2.22 ± 0.12 kg *vs.* brood mass in control group = 3.80 ± 0.43 kg, *W* = 36, *P* = 0.035). The surviving chicks were not affected by the treatment of the male, because there were no differences in their body masses and flipper lengths compared with control chicks (Table 2, Fig. 5). Indeed, chick mass was not significantly different between control and CORT-treated birds at the end of the experiment, i.e. 21–31 days after hatching (Wald χ^2^ = 0.002, *P* = 0.969) and 39–43 days after hatching (Wald χ^2^ = 1.393, *P* = 0.238). Brood mass did not differ between groups after the experiment, when chicks were 39–43 days old (*W* = 31, *P* = 0.181).

**Table 1:** profiles of control and CORT-treated male Adélie penguins and hatching date of the first chick

	Control	CORT	*W*	*P*-value
*n*	7	7		
Body mass (kg)	4.692 ± 0.172	4.703 ± 0.149	25	1
Flipper length (cm)	19.16 ± 0.27	19.56 ± 0.21	15	0.248
Scaled mass index	4.69 ± 0.14	4.71 ± 0.17	26	0.902
Hatching date of first chick	22 Dec ± 0.96	24 Dec ± 0.59	12	0.119

Results are expressed as means ± SEM. Abbreviation: CORT, corticosterone. See text for further details.

**Table 2:** profiles of chicks from control and CORT-treated Adélie penguins throughout the study period

	Control	CORT	Wald χ^2^	*P*-value
Chicks at 21–31 days				
*n* (*n*_chicks_/*n*_pairs_)	12/7	7/7		
Date	16 Jan ± 0.3	17 Jan ± 0.3	1.215	0.270
Age (days)	25.1 ± 0.9	24.0 ± 0.6	0.628	0.428
Body mass (kg)	2.13 ± 0.12	2.22 ± 0.13	0.002	0.969
Flipper length (cm)	15.5 ± 0.5	15.3 ± 0.6	0.073	0.787
Chicks at 39–43 days				
*n* (*n*_chicks_/*n*_pairs_)	11/7	7/7		
Date	1 Feb ± 0.8	3 Feb ± 0.7	2.678	0.102
Age (days)	41.2 ± 0.4	41.8 ± 0.4	2.022	0.155
Body mass (kg)	2.88 ± 0.18	3.27 ± 0.30	1.393	0.238
Flipper length (cm)	18.6 ± 0.3	18.7 ± 0.5	0.120	0.729

Results are expressed as means ± SEM. See text for further details.

There was also no relationship between the body mass of adult males and brood mass in both groups at the end of the experiment (Spearman correlation, control, *P* = 0.333 and CORT, *P* = 1) and between adult scaled mass indexes and brood mass (Spearman correlation, control, *P* = 0.302 and CORT, *P* = 1).

### Composition of the diet

**Figure 6:**
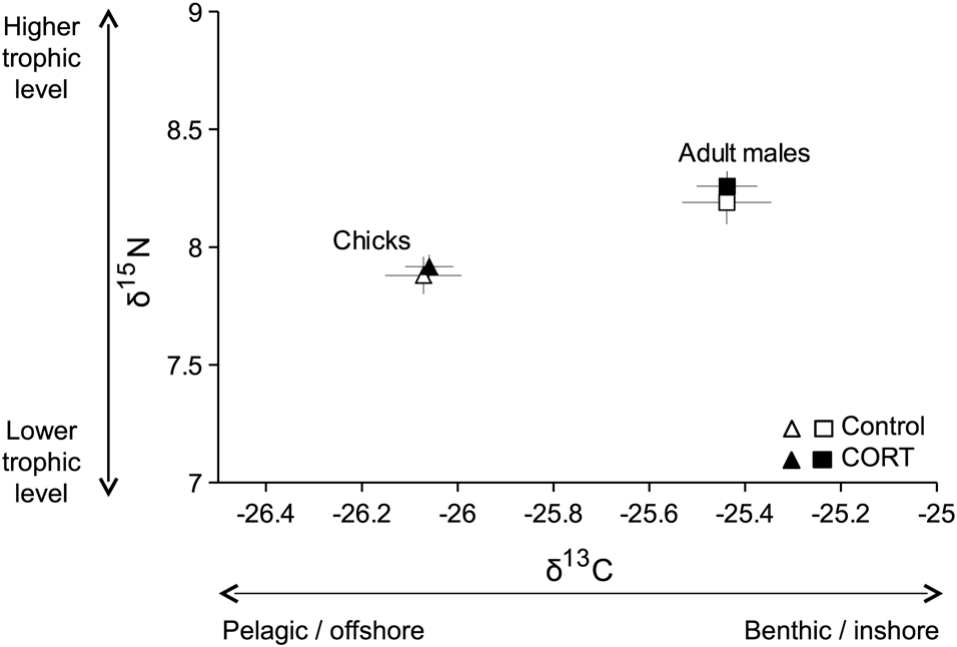
δ^15^N and δ^13^C values (means ± SEM) of CORT-treated and control male Adélie penguins and their chicks during the guard stage.

The overall isotopic signature of control and CORT-treated adult males (Fig. 6; MANOVA, *F*_2_ = 0.123, *P* = 0.885) and that of their respective chicks (*F*_2_ = 0.018, *P* = 0.982) did not differ significantly. However, there were differences in overall isotopic signatures between adults and chicks (*F*_2_ = 36.906, *P* < 0.001; Fig. 6). Control adult males exhibited higher δ^13^C values than their chicks (control adult males, δ^13^C, −25.44 ± 0.09 ‰ and control chicks, δ^13^C, −26.07 ± 0.08 ‰, *W* = 61, *P* = 0.002). There were no significant differences of δ^15^N values between chicks and adult males (*W* = 47, *P* = 0.114). Similar trends were obtained between CORT-treated adult males and their chicks (δ^13^C, *W* = 35, *P* = 0.003 and δ^15^N, *W* = 27, *P* = 0.141).

## Discussion

In this study, we investigated the effects of increased CORT levels on the breeding effort and the reproductive output of chick-rearing male Adélie penguins.

### Experimental manipulation of CORT levels

Small quantities of blood were sampled in the present study to minimize handling time and disturbance. These small samples were not sufficient for CORT assays to be performed. However, whether the treatment increased CORT levels within baseline ranges or to acute stress-induced levels, both within the normal physiological range, may have different effects (Landys *et al.*, 2006). Corticosterone levels of treated chick-rearing male Adélie penguins were measured in a similar experiment with the same study protocol (Thierry A.M. and Raclot T., unpublished data). Blood was sampled on the day of implantation and an average of 17.9 ± 0.2 days after pellet implantation and plasma kept for CORT assay. Pre-treatment CORT levels did not differ between groups (3.9 ± 1.0 and 4.7 ± 0.5 ng/ml for control birds and CORT-treated birds, respectively). Corticosterone levels had returned to initial levels by the time of the second blood sample (4.9 ± 0.5 and 4.6 ± 0.8 ng/ml for control birds and CORT-treated birds, respectively, about 18 days post-implantation). The return to baseline levels 18 days after CORT implantation is also in agreement with the study of Spée *et al.* (2011a) using similar pellets in Adélie penguins. A 2- to 4-fold increase in CORT levels was measured in CORT-treated incubating male Adélie penguins (Spée *et al.*, 2011a; Thierry *et al.*, 2013), i.e. below the the levels reached during capture stress (Cockrem *et al.*, 2009), and within the physiological range of this species.

Corticosterone pellets have been described to induce a peak-like elevation of circulating CORT levels and temporarily to remove the endogenous response to an acute stressor (Müller *et al.*, 2009). In particular, highly elevated levels of CORT trigger different responses compared with slightly elevated levels (Wingfield *et al.*, 1997, 1998). Intermediate CORT levels may increase food searching in a free-living animal, while higher levels may restrict movement (Breuner *et al.*, 1998; Breuner and Wingfield, 2000; Crossin *et al.*, 2012). In the present study, the lack of marked changes in the behaviour of male Adélie penguins on land (Raclot T., visual observations) suggests that CORT did not drastically affect the time budget of birds. Self-degradable pellets remain an efficient tool to elevate hormone levels experimentally in field studies.

Whether baseline and/or stress-induced CORT levels are good predictors of fitness in free-living animals is not yet clear (Breuner *et al.*, 2008; Bonier *et al.*, 2009a). A negative relationship between baseline glucocorticoid levels and fitness measures, mediated by environmental conditions or challenges, is described under the CORT-fitness hypothesis, with partial empirical support. Nevertheless, baseline CORT levels vary consistently among individuals, depending on environmental conditions and life-history stages, making a unidirectional link between CORT and fitness unlikely (Dingemanse *et al.*, 2010). The CORT-adaptation hypothesis expands the definition of environmental challenges to include challenges associated with reproduction (Bonier *et al.*, 2009b), which predicts that increased reproductive demand causes increases in baseline CORT levels. The nature of the relationship between CORT and fitness, and the validity of the CORT-adaptation hypothesis, depend on the magnitude of reproductive investment, and may thus vary among species and individuals with different reproductive strategies. Understanding how CORT relates to fitness becomes even more complex when considering that behaviour, size, and morphology also affect how organisms perceive environmental signals (Kearney, 2006), and may therefore affect CORT levels.

### Corticosterone and time spent at the nest

Negative effects of elevated CORT levels on parental care in birds have been described in many studies (Silverin, 1986; Wingfield and Sapolsky, 2003; Criscuolo *et al.*, 2006; Almasi *et al.*, 2008; Groscolas *et al.*, 2008; Angelier *et al.*, 2009; Horton and Holberton, 2009; Thierry *et al.*, 2013). However, the CORT-adaptation hypothesis predicts that high CORT levels support increased foraging activity and parental effort (Bonier *et al.*, 2009a). Indeed, increased CORT levels induced an increase in foraging activity and parental care in female macaroni penguins (Crossin *et al.*, 2012). Interestingly, CORT-treated male Adélie penguins had a decreased reproductive output, while spending more time at their nest. Their females apparently did not compensate for this, because they did not modify their time budget. Likewise, female Adélie penguins were not affected by the temporary handicapping of their partner (forcing them to conduct longer trips), and had a decreased reproductive output (Beaulieu *et al.*, 2009).

Corticosterone treatment is known to have a negative effect on body mass, which can be explained, at least partly, by a shift in fuel utilization (Spée *et al.*, 2011b). Although CORT-treated penguins fasted for longer while at the nest, they maintained a similar body condition to that of control birds and provided enough food to their remaining chicks, which had similar body mass and size to control chicks (Table 1). Given that CORT-treated birds had a lower number of chicks to supply and knowing that foraging at sea is more costly than guarding the chicks (Chappell *et al.*, 1993), spending more time at the nest probably allowed CORT-treated birds to maintain a similar body mass to that of control birds. Our results support the idea that increasing CORT levels may have induced birds to allocate the available energy to the benefit of body maintenance at the expense of current reproduction.

Corticosterone-treated penguins had a 42% decrease in reproductive output, but all birds raised at least one chick. Criscuolo *et al.* (2005) found that exogenous CORT increased the rate of egg loss of incubating female common eiders (*Somateria mollissima*) through a reduction in nest attentiveness associated with decreased prolactin levels. Corticosterone-treated chick-rearing Adélie penguins may have been less attentive to their nest, leading to less efficient protection of the chicks against cold or predators. Visual observations in the field do not support this idea, however, because predators are few on this part of the Dumont d'Urville colony and adverse weather did not occur much during the study period.

It is possible that the quality or quantity of chick provisioning may have been reduced in CORT-treated birds. Horton and Holberton (2009) found an inhibitory effect of elevated CORT levels on chick-provisioning frequency in male white-throated sparrows (*Zonotrichia albicollis*). Given that the growth rate of Adélie penguin chicks is positively affected by the feeding frequency (Takahashi *et al.*, 2003), the lower reproductive output of CORT-treated males was probably caused by a reduced chick-provisioning rate.

Further studies are needed to examine the timing and the causes of chick mortality (e.g. by using video recording and regular weighing of the chicks) in treated birds in order to understand better how increased CORT levels affect reproductive output.

### Corticosterone and foraging trips

While CORT-treated penguins spent more time at their nest, they did not spend more time foraging at sea. Likewise, CORT treatment did not affect foraging trip durations of female macaroni penguins relative to control birds, but diving behaviour differed between the two groups (Crossin *et al.*, 2012). Telemetry showed that dive parameters, such as the number of dives, the mean depth, and the number of foraging events per dive, differed between control and CORT-treated birds, with an overall positive effect of the CORT treatment on foraging behaviour and diving activity, independent of the time spent away from the colony on trips. Although we did not study the foraging behaviour of our birds in the present study, we may expect the CORT treatment to affect the birds' foraging behaviour as suggested by a preliminary study using time–depth recorders on CORT-implanted Adélie penguins (Cottin *et al.*, 2011). As for Crossin *et al.* (2012), Adélie penguins implanted with CORT pellets seemed to increase their diving effort, although trip duration did not change (Cottin *et al.*, 2011).

In a recent correlative study, Angelier *et al.* (2008) showed that unmanipulated Adélie penguins with elevated pre-trip CORT levels spent less time at sea and foraged closer to the colony in comparison to individuals with low CORT levels. As elevated CORT levels were associated with active (high-effort) short trips and led to a low mass gain, the authors suggested that elevated CORT levels may help the birds to support the increased energetic demands associated with chick rearing by stimulating foraging activity at the expense of the adult body reserves. On the contrary, an experimental elevation of baseline CORT levels led treated black-legged kittiwakes to increase their foraging activities at the expense of guarding their chicks at the nest (Kitaysky *et al.*, 2001). These different studies highlight the complexity of the physiological mechanisms driving changes in foraging behaviour among species. Four non-exclusive explanations can be put forward to explain the absence of an effect of CORT on foraging duration, as follows.

- As mentioned above, the trip duration is not affected, but foraging parameters within the foraging trip window may be modified (see Cottin *et al.*, 2011; Crossin *et al.*, 2012), i.e. regulation takes place at the level of the diving effort but not at the larger-scale level of the foraging trip. This seems plausible because a CORT increase induces an increase in locomotor (corresponding here to diving) activity (Spée *et al.*, 2011b).
- Corticosterone has an inverted U-shaped dose–response curve; only intermediate levels of CORT activate behaviour, while low and high levels have no effect (Breuner and Wingfield, 2000). The CORT pellets used in our study were found to mimic metabolic, hormonal, and behavioural changes of long-term fasting in birds (Spée *et al.*, 2011b). In that study, there was a 2.5-fold increase in locomotor activity shortly after implantation in the failed breeders kept in captivity and treated with 100 mg of CORT, as in our study. Nevertheless, the CORT dose may have been too high to affect foraging behaviour and did not seem to affect birds' behaviour at the nest (Raclot T., personal observations).
- The effects of CORT on foraging behaviour might depend on the nutritional status of the birds. For example, CORT implantation in fed white-crowned sparrows (*Zonotrichia leucophrys*) did not affect food intake, while fasting CORT-implanted birds increased their foraging activity (Astheimer *et al.*, 1992). In our study, the nutritional status of birds was difficult to assess. Although birds were regularly feeding at sea, they regurgitated part of the food they ingested to feed their chicks. However, Adélie penguins can fast for several weeks before they reach a low threshold in their body reserves, which precedes nest abandonment to refeed at sea (Spée *et al.*, 2010). Thus, it is likely that CORT-treated birds did not reach a late stage of fasting in this study.
- Other physiological mechanisms, such as negative feedback processes, the type and distribution of receptors within target tissues, and the concentration of corticosteroid-binding globulins (Almasi *et al.*, 2009), may have prevented effects of exogenous CORT on foraging trip duration. Other endocrine factors could also modulate foraging trip durations. For example, prolactin, the main hormone involved in parental care in birds (Buntin, 1996), has recently been suggested to be involved in the mediation of the trade-off between the reproductive effort and self-maintenance (Angelier and Chastel, 2009). Corticosterone may indirectly affect this trade-off via a stress-induced effect on prolactin, which would then affect the trade-off between chick provisioning and self-maintenance. Besides, recent studies suggest that CORT could affect prolactin levels more directly (Criscuolo *et al.*, 2005; Angelier *et al.*, 2009; Spée *et al.*, 2011a), although such a link is not found in all species (e.g. Crossin *et al.*, 2012).

### Conclusions and perspectives

We showed that experimentally elevated CORT levels increased the time that birds spent at their nest, but did not affect foraging trip durations, foraging sites, and diet quality in terms of isotopic signature. Interestingly, while the treatment decreased reproductive output (the number of chicks fledged per nest), it did not affect the growth of the surviving chicks.

The detailed behaviour of treated birds should be examined in further studies to provide understanding of how elevated baseline CORT levels increased chick mortality. Given that seabirds are central-place foragers, feeding at sea and foraging activities also need to be monitored to test whether CORT-treated individuals could be more or less efficient in terms of prey capture, and thus more or less efficient in terms of chick provisioning.

Birds have been used extensively as models to explore the ecological basis of stress and the underlying endocrine mechanisms. Ecologists and conservation biologists also use levels of stress hormones as an indicator of physiological stress in wild animals, and in extension, as a correlate of the ‘health status’ of a population. Nevertheless, the relationships between glucocorticoids, fitness, and ultimately population dynamics are not fully understood and remain controversial depending on species, constraints, and changes in the environment. Studies of stress hormones using both correlative and experimental approaches are of great interest and are fundamental in order for conservation physiology to be successful as a discipline, helping to manage species of conservation concern facing detrimental conditions in a rapidly changing environment.
